# Leukemia cell microvesicles promote survival in umbilical cord blood hematopoietic stem cells

**DOI:** 10.17179/excli2015-101

**Published:** 2015-03-13

**Authors:** Farnaz Razmkhah, Masoud Soleimani, Davood Mehrabani, Mohammad Hossein Karimi, Sedigheh Amini Kafi-abad

**Affiliations:** 1 Department of Hematology, Faculty of Medical Sciences, Tarbiat Modares University, Tehran, Iran; 2 Transgenic Technology Research Center, Shiraz University of Medical Sciences, Shiraz, Iran; 3 Transplant Research Center, Shiraz University of Medical Sciences, Shiraz, Iran; 4 Department of Pathology, Blood Transfusion Research Center, High Institute for Research and Education in Transfusion Medicine, Tehran, Iran

**Keywords:** microvesicles, cell survival, leukemia, hematopoietic stem cells, P53 gene expression

## Abstract

Microvesicles can transfer their contents, proteins and RNA, to target cells and thereby transform them. This may induce apoptosis or survival depending on cell origin and the target cell. In this study, we investigate the effect of leukemic cell microvesicles on umbilical cord blood hematopoietic stem cells to seek evidence of apoptosis or cell survival. Microvesicles were isolated from both healthy donor bone marrow samples and Jurkat cells by ultra-centrifugation and were added to hematopoietic stem cells sorted from umbilical cord blood samples by magnetic associated cell sorting (MACS) technique. After 7 days, cell count, cell viability, flow cytometry analysis for hematopoietic stem cell markers and qPCR for P53 gene expression were performed. The results showed higher cell number, higher cell viability rate and lower P53 gene expression in leukemia group in comparison with normal and control groups. Also, CD34 expression as the most important hematopoietic stem cell marker, did not change during the treatment and lineage differentiation was not observed. In conclusion, this study showed anti-apoptotic effect of leukemia cell derived microvesicles on umbilical cord blood hematopoietic stem cells.

## Introduction

Microvesicles are small (0.05-1 µm) membrane-bound particles released by different cells including both healthy and tumor types (Taylor and Gercel-Taylor, 2011[25]; Mathivanan et al., 2010[15]; Camussi et al., 2010[6]). They contain proteins, different kinds of RNA, lipids and organelles from the cells of origin to activate target cells, directly (Skog et al., 2008[24]; Mitchell et al., 2008[16]; Gilad et al., 2008[11]).

Microvesicles are involved in different (patho)physiological processes in the body such as cell survival (Hussein et al., 2007[14]; Shedden et al., 2003[23]), escape from the immune system (Whitlow and Klein, 1997[28]; Hakulinen et al., 2004[12]), angiogenesis (Tesselaar et al., 2007[26]; Rauch and Antoniak, 2007[21]) and metastasis (Al-Nedawi et al., 2008[1]; Skog et al., 2008[24]). These effects are based on *a)* microvesicles' specific receptor/ligand interactions with target cells (Chalmin et al., 2010[7]; Nolte-'t Hoen et al., 2009[17]), *b)* transferring cell surface receptors (Baj-Krzyworzeka et al., 2006[3]) and *c)* intracellular proteins and RNAs delivery into recipient cells (Putz et al., 2012[19]; Zhang et al., 2010[29]).

Different studies focused on target cells transformation by microvesicles, have shown acquisition of aggressive cancerous phenotypes by a non-aggressive population of tumor cells through microvesicles (Al-Nedawi et al., 2008[1]), transformation of normal hematopoietic transplants through genomic instability induced by BCR-ABL positive microvesicles (Zhu et al., 2014[30]) and reprogramming of hematopoietic progenitors by embryonic stem cells derived microvesicles (Ratajczak et al., 2006[20]).

Based on these findings, we chose umbilical cord blood hematopoietic stem cells, as a remarkable source for stem cell transplantation, to be our target cell for leukemia cell microvesicles. We were interested to see whether these microvesicles have any affect hematopoietic stem cell survival or cause apoptosis. 

## Materials and Methods

### Human samples and cell line preparation

Bone marrow aspiration was obtained from healthy volunteers (written informed consent was obtained) in accordance with ethical standards of the responsible committee on human experimentation in Tarbiat Modares University. Red blood cell lysis was done by ammonium chloride and the remaining cells were used for microvesicles isolation.

Jurkat cells were maintained in RPMI 1640 containing 15 % fetal bovine serum, 100 U/ml penicillin and 100 µg/ml streptomycin at 37 °C, 5 % CO_2_ and 90 % humidity.

### Microvesicles isolation

Both normal bone marrow cells and Jurkat cells were transferred to RPMI 1640 supplemented with 0.6 % bovine serum albumin overnight. Then, the supernatant was collected and freshly used. Cell-free supernatants were obtained by a 2,000 rpm centrifugation at 4 °C for 10 minutes. Cell debris and apoptotic bodies were excluded by 10,000 g centrifugation at 4 °C for 20 minutes. Macrovesicles pallets were achieved after 20,000 g centrifugation at 4 °C for one hour and finally, the microvesicles were washed in phosphate buffered saline after repeated centrifugation at 20,000 g. The pellets were used freshly for both calculating protein concentration by Bradford assay and co-incubation with target cells.

### Microvesicles transmission electron microscopy

Isolated microvesicles were stained with 2 % -uranyl acetate on formvar-carbon coated grids as a negative stain. After drying, the grid was placed in electron microscope to provide transmission images. 

### Hematopoietic stem cells sorting

Umbilical cord blood samples were obtained from Iranian blood Transfusion Organization in CPDA1 reagent (written consent was obtained). MACS technique was used to sort hematopoietic stem cells by CD34 magnetic immunobeads (Milteny Biotec, Auburn, CA). Purity of the cells was analyzed by flow cytometry.

### Co-incubation of hematopoietic stem cells and isolated Microvesicles

55,000 sorted hematopoietic stem cells were treated with 20 µg/ml microvesicles from normal bone marrow cells (normal group) and Jurkat cells (leukemia group) in 500 µl Stemline medium (Sigma-Aldrich) containing 50 ng/ml TPO (Pepro Tech) and FLT3 (ORF Genetics) recombinant proteins and were kept at 37 °C, 5 % CO_2_ and 90 % humidity for 7 days. In the control group, cells were incubated without any microvesicle.

### Cell count

After 7 days, the number of cells was evaluated in a hemocytometer chamber by using Trypan Blue.

### Flow cytometry analysis

Cell viability was analyzed by 7AAD (PE-Texas Red, Sigma-Aldrich). CD34 (PE, eBiosciences) and CD45 (FITC, BD) antibodies were used for cell staining to evaluate purity and also hematopoietic stem cell markers. CD2 and CD19 antibodies (both BD) were used to show lineage differentiation.

### Quantitative real time PCR

Total RNA was extracted using RNX Plus reagent (Sinagen, Iran) and cDNA was synthesized according to the instruction (Fermentase). Expression of P53, as a cell cycle gene and HPRT as a housekeeping gene, was analyzed by real time detection system (Applied Biosystems Plus one) using SYBR Green master mix (Takara, Japan) according to the manufacturer's protocol. Both genes' primer sequences are provided in Table 1(Tab. 1). Relative gene expression fold change was calculated with 2(‐∆∆Ct) formula.

### Statistical analysis

All experiments were replicated three times, independently and data was presented as mean ± SD. SPSS 22 software was used for one-way ANOVA analysis (for normal distributions) and Kruskal-Wallis test (for abnormal distributions). P<0.05 was considered statistically significant.

## Results

### Transmission electron microscopy image confirming the size of isolated microvesicles

**Figure 1:**
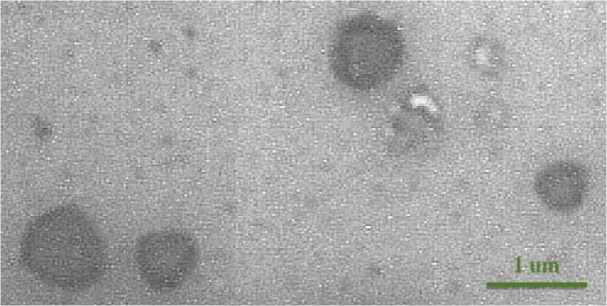
Transmission electron microscopy image of isolated microvesicles (6000X)

As shown in Figure 1(Fig. 1), microvesicles' size on electron microscopy was less than 1 µm, indicating the true protocol for isolation and purification of microvesicles with a size range of 0.1-1 µm.

### Jurkat microvesicles retain the number of hematopoietic stem cells 

**Figure 2:**
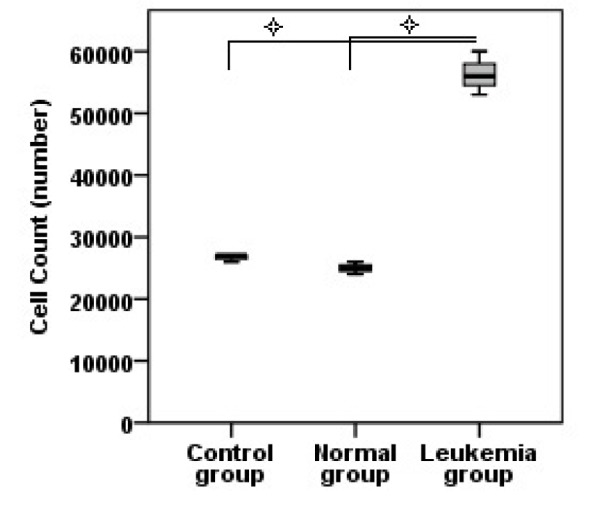
Cell count. Values are mean ± SD of three samples in each group

After 7 days, the number of hematopoietic stem cells treated with Jurkat microvesicles was more than that in the other two groups (Figure 2(Fig. 2)) and this difference was statistically significant (P<0.05). However, the number of cells in control and normal groups was less than the first day seeding number. 

### Jurkat microvesicles keep hematopoietic stem cells alive

**Figure 3:**
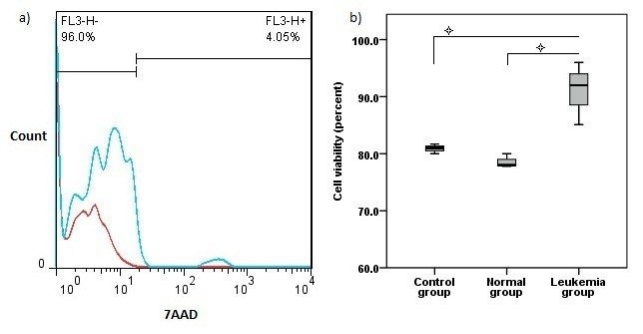
Cell viability test. a) 7AAD staining results with blue histogram show one sample from leukemia group merged with red histogram. b) Viable cells values in three studied groups (mean ± SD).

Flow cytometry analysis showed a higher range of viability in leukemia group in comparison with control and normal groups (P<0.05) (Figure 3a, b(Fig. 3)). 

### Jurkat microvesicles do not change hematopoietic stem cell markers

**Figure 4:**
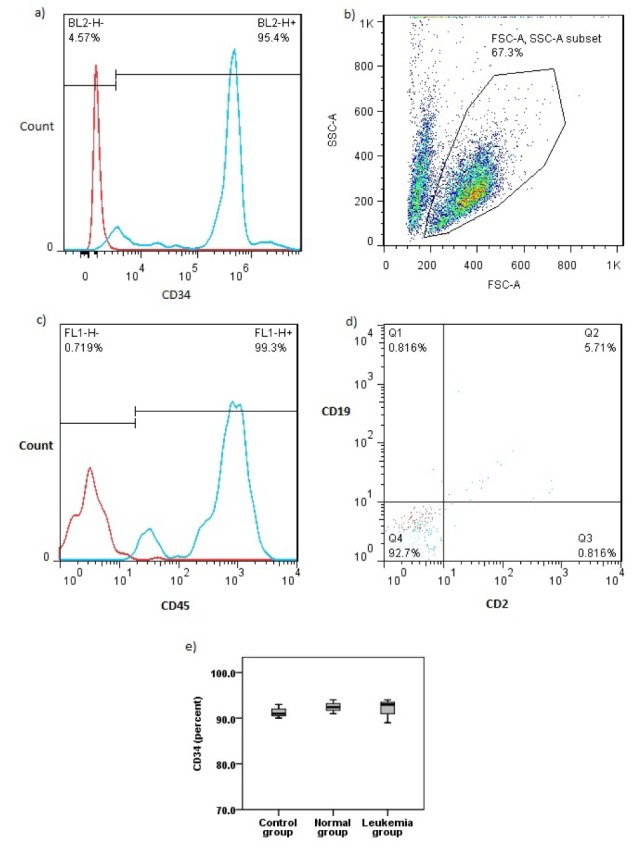
Flow cytometry analysis for hematopoietic stem cell markers. a) Purity of first day sorted hematopoietic stem cells. b) Gated population. c) CD45 expression after treatment. d) CD2 and CD19 expression after treatment. e) CD 34 expression after treatment in all studied groups.

CD34 expression was not significantly different among studied groups (P>0.05) and was close to CD34 expression in the first day sorted hematopoietic stem cells. CD45 expression was still high. CD2 and CD19 expression were both in minimum range showing no differentiation in stem cells during the treatment with microvesicles (Figure 4a-e(Fig. 4)).

### Jurkat microvesicles decrease P53 gene expression

**Figure 5:**
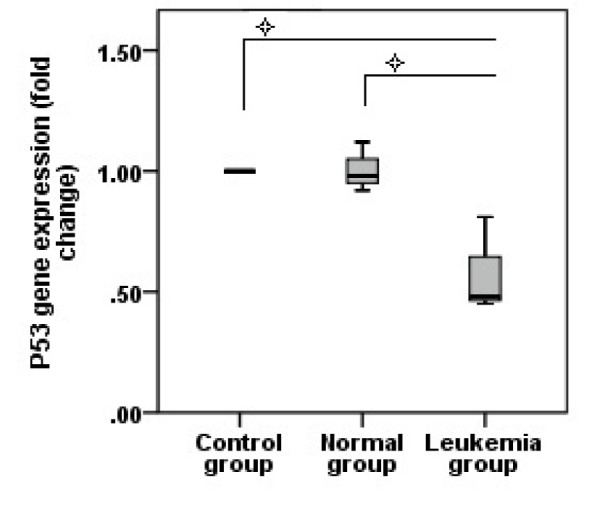
P53 gene expression fold change in studied groups after treating with microvesicles

Quantitative real time PCR showed a decrease in P53 gene expression, normalized with HPRT, compared to control and normal groups (P=0.01) (Figure 5(Fig. 5)). 

## Discussion

Microvesicles take part in numerous physiological events. Behind of intercellular communications, as one of the most important functions, they are also involved in regulation of programmed cell death (Hussein et al., 2007[14]; Furie and Furie, 2008[10]; Distler et al., 2005[8]). Generally, cells shed microvesicles to protect against intracellular stress (Doormal et al., 2009[9]). Microvesicles containing caspase3, one of the main executioner enzymes of apoptosis, are present in conditioned medium of viable cell culture; but this enzyme is not detectable in those cells microvesicles originated from (Boing et al., 2008[5]; Sapet et al., 2006[22]). Thus, cells may escape from apoptosis by releasing these microvesicles. On the other hand, some studies especially the one by Valenti and colleagues showed the anti-apoptotic effect of tumor cell microvesicles on target cells. They showed that CD14+ cells from peripheral blood were resistant to apoptosis induced by microvesicles released from tumor cells such as melanoma and colorectal carcinoma cell lines (Valenti et al., 2006[27]). However, previously they showed apoptosis induction in activated T cells (Jurkat) by tumor cells microvesicles (Andreola et al., 2002[2]; Huber et al., 2005[13]). Also, another study showed a decreased level of P53 gene expression in mono nuclear cells treated with leukemia cell microvesicles (K562) (Zhu et al., 2014[30]). The ability of P53 to promote cell cycle arrest and apoptosis is well known and appears to induce apoptosis by multiple pathways (Benchimol, 2001[4]). In concordance with the above-mentioned research, we showed the anti-apoptotic effect of leukemia cell microvesicles on target cells. Our results indicated lower level of P53 gene expression in umbilical cord blood hematopoietic stem cells treated with Jurkat cell microvesicles in comparison with control and normal groups. 

In addition, this study is the first investigation seeking the effect of leukemic cell microvesicles directly on umbilical cord blood hematopoietic stem cells which are among the most important sources for stem cell transplantation. We showed high viability and survival of these cells when compared with other groups which was close to the seeding number on day 0. These results were much more brilliant when we observed a high expression of CD34 and CD45 markers but not lineage markers such as CD2 and CD19, indicating that umbilical cord blood hematopoietic stem cells, which were treated with Jurkat cell microvesicles, expressed important hematopoietic stem cell markers after 7 days. Thus, we can conclude that this treatment is not able to promote differentiation in studied stem cells. 

We designed our experiment based on the role of microvesicles in inducing potential effects on target cells. To achieve this goal, the conditioned medium for treating hematopoietic stem cells with microvesicles, included FLT3 and TPO cytokines and lacked Stem Cell Factor according to Piacibello's study (Piacibello et al., 1997[18]) to have the fewest level of expansion and the highest level of colony formation during the first two weeks. It was the most important check point in our work to remark microvesicles power to show their effects. 

In summary, we showed that Jurkat cell microvesicles are able to induce apoptosis resistance in umbilical cord blood hematopoietic stem cells. The results indicated reasonably viable number of hematopoietic stem cells in presence of leukemia microvesicles which still expressed more than 90 % CD34 and CD45 markers without differentiation. 

## Acknowledgements

This study was supported by the Tarbiat Modares University. We would like to thank Iranian Blood Transfusion Organization for providing umbilical cord blood samples and Taleghani Hospital for healthy donor bone marrow samples. We also acknowledge the stem cell technology research center for all the equipment and technical supports.

## Conflict of interest

The authors declare no conflict of interest.

## Figures and Tables

**Table 1:**
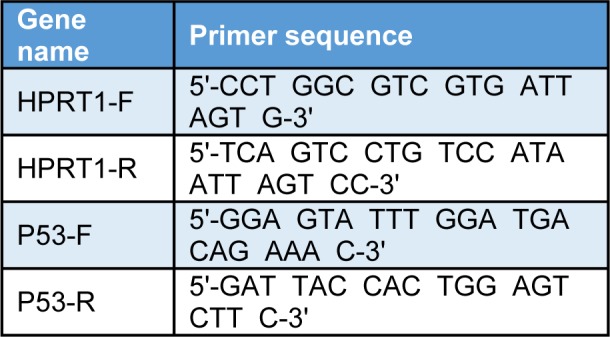
Table1: Primer sequences
